# Can Platelet-Rich Plasma Reduce the Burden of Inflammatory Skin Diseases Such as Psoriasis and Atopic Dermatitis?

**DOI:** 10.7759/cureus.18472

**Published:** 2021-10-04

**Authors:** Winfried Kauhl, Hanno Pototschnig, Uwe Paasch

**Affiliations:** 1 Plastic and Reconstructive Surgery, Gemeinschaftspraxis Dr. med. Kauhl Dr. med. Cymorek, Moenchengladbach, DEU; 2 Regenerative Medicine, Munich Medical Esthetic, Munich, DEU; 3 Department of Dermatology, Venereology, and Allergology, University of Leipzig Medical Center, Leipzig, DEU

**Keywords:** platelet-rich plasma, prp, acp, psoriasis, atopic dermatitis, atopic eczema

## Abstract

Objective

In this study, our aim was to investigate the clinical effects of platelet-rich plasma (PRP) on the skin of patients suffering from plaque psoriasis or atopic dermatitis.

Methods

Over a period of 53 months, we treated a total of 40 patients for inflammatory skin diseases with PRP. All of these patients were included in this study; 5-6 ml of PRP were prepared with the autologous-conditioned plasma (ACP) double syringe and injected subdermally. Follow-ups were conducted at three, six, nine, and 12 weeks after treatment. Besides the lesion size, Psoriasis Area and Severity Index (PASI) and Eczema Area and Severity Index (EASI) were also calculated. Data were evaluated statistically at a significance level of p≤0.05.

Results

A total of 30 patients were treated for plaque psoriasis. The elbow area represented the most common area of treatment (17 cases). The average lesion size decreased from 8.2 cm² to 0.3 cm² (p<0.00001). Of note, 80% of all patients achieved complete remission (PASI100) at the last follow-up. The remaining 20% reached at least PASI70. Ten patients were treated for atopic dermatitis. In six cases, efflorescences on patients’ arms were treated, and in four cases, patients' legs were treated. The average lesion size decreased from 8 cm² to 0.155 cm² (p<0.00001). Notably, 50% of all patients achieved complete remission (EASI100) at the last follow-up. The other half reached at least EASI70. In all cases, the lesion size decreased progressively. No adverse events were reported.

Conclusion

Our study revealed encouraging results for both psoriasis and atopic dermatitis. The autologous treatment was safe and effective in all patients. Further studies are required to validate these initial findings.

## Introduction

With a prevalence of 2-4% and 7% respectively, psoriasis vulgaris and atopic eczema are widespread chronic inflammatory skin diseases, which are associated with a huge impairment in the quality of life of patients [1,2]. To reduce the negative impact on patients’ quality of life, numerous systemic treatment modalities have been introduced recently, specifically for severe types of these diseases. However, there is still a need for limited courses where the latest systemic therapies might not be applicable [3]. Platelet-rich plasma (PRP) has become a promising treatment modality in aesthetic dermatology, trichology, and wound care [4-6]. Regarding its application for skin rejuvenation, Maisel-Campbell et al. recently reviewed 24 studies, including eight randomized controlled trials (RCTs), and concluded that PRP injections are safe and there is convincing evidence for the improvement of facial skin texture following their administration [4]. Regarding the treatment of androgenetic alopecia (AGA), Chen et al. reviewed eight RCTs and 16 prospective cohort studies systemically. They found that 21 studies reported positive outcomes by objective criteria (88%), and no serious adverse events were reported. The authors concluded that PRP is a low-risk intervention to treat AGA, and it is associated with good patient satisfaction and objective improvements in outcomes [5]. Hu et al. recently published a systematic review and meta-analysis that reviewed eight RCTs and stated that PRP may improve ulcer healing without significant adverse effects in patients with diabetic ulcers [6]. However, evidence regarding the treatment of psoriasis or atopic dermatitis is currently limited to one study that has reported encouraging results, especially the adjunct treatment of plaque psoriasis in combination with methotrexate (MTX) [7]. Since PRP treatment is effective in a wide variety of skin conditions where chronic inflammation is known to be the underlying mechanism of action, it is assumed that PRP might also be useful in atopic dermatitis and psoriasis. In light of this, our aim was to investigate the clinical effects of a single PRP injection on the skin of patients suffering from limited types of plaque psoriasis or atopic dermatitis.

## Materials and methods

Over a period of 53 months, we treated a total of 40 patients with a single injection of PRP for inflammatory skin diseases. All of these patients were included in this retrospective study. The principles outlined in the Declaration of Helsinki were followed; consent was obtained from all participants. Treatments were performed as part of the daily routine. For the preparation of PRP, the autologous-conditioned plasma (ACP) double syringe (Arthrex Inc., Naples, FL) was spun for five minutes at approximately 350 G by using a Rotofix 32A swing-out rotor centrifuge (Andreas Hettich GmbH & Co. KG, Tuttlingen, Germany). According to hematological analyses, PRP prepared with this system is peculiarly poor in leukocytes (especially neutrophils) and erythrocytes, and it has an average platelet concentration of 2.42-fold over baseline [8]. After local disinfection, local anesthesia was administered using a numbing cream with occlusion patches; 5-6 ml of PRP was applied by subdermal injections. During consecutive clinical investigations [three (T1), six (T2), nine (T3), and 12 (T4) weeks after the treatment], photographs were taken for documentation and the treating physician assessed the size of the lesions and the quality of the skin efflorescences. Psoriasis Area and Severity Index (PASI) and Eczema Area and Severity Index (EASI) were respectively calculated retrospectively for the status before treatment and at the last follow-up. Data were evaluated statistically using the Mann-Whitney U test; the significance level was set at a p-value of ≤0.05.

## Results

**Figure 1:**
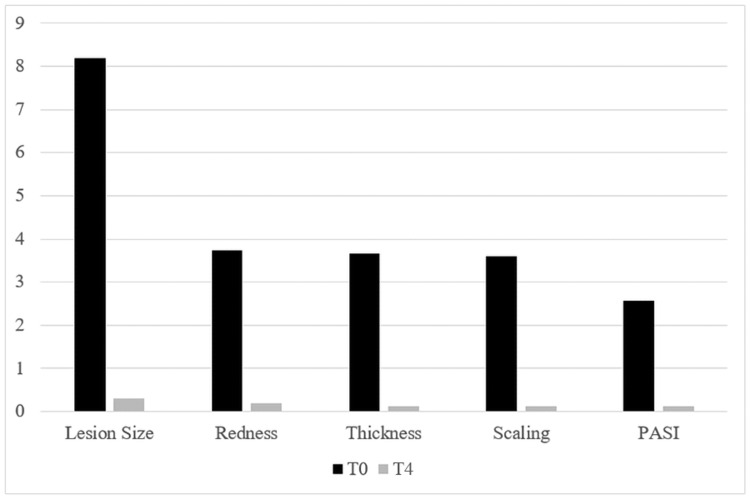
Signs of psoriasis before treatment (T0) and improvement at the three-month follow-up (T4) PASI: Psoriasis Area and Severity Index

A total of 30 patients with an average age of 52 years were treated for localized plaque psoriasis. Of them, 23 were females and seven were males. The elbow area was the most frequent area of treatment (17 cases). Lesions on the trunk were treated in six cases, lesions on the head in four cases, and lesions on the legs in three cases. The average lesion size decreased significantly from 8.2 cm² to 0.3 cm² (p<0.00001). Clinical improvements and the reduction of the PASI (p<0.00001) are presented in Figure 1.

**Figure 2:**
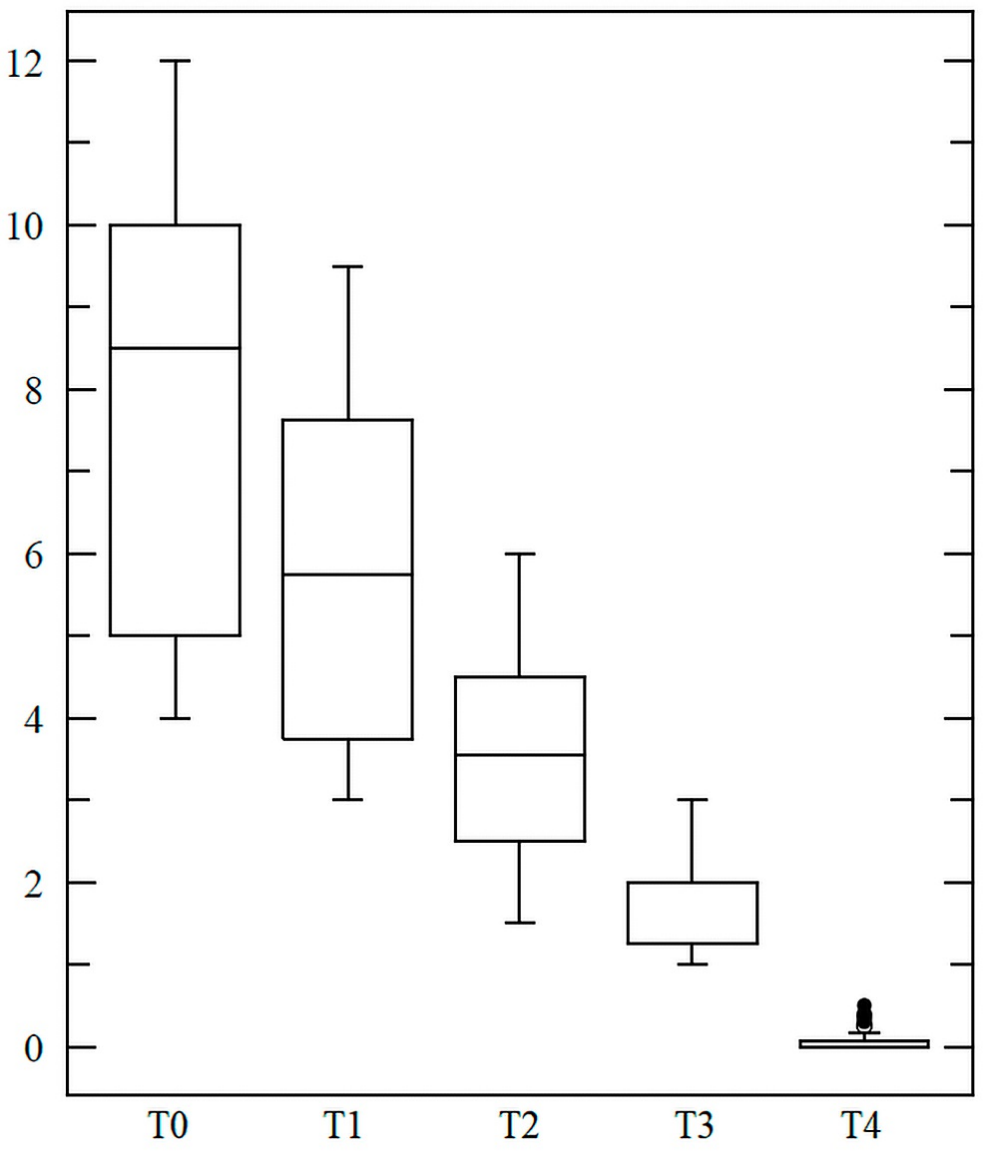
Progressive reduction in psoriasis lesion size (cm²) The image shows lesion size before treatment (T0), three weeks after (T1), six weeks after (T2), nine weeks after (T3), and at the three-month follow-up (T4)

**Figure 3:**
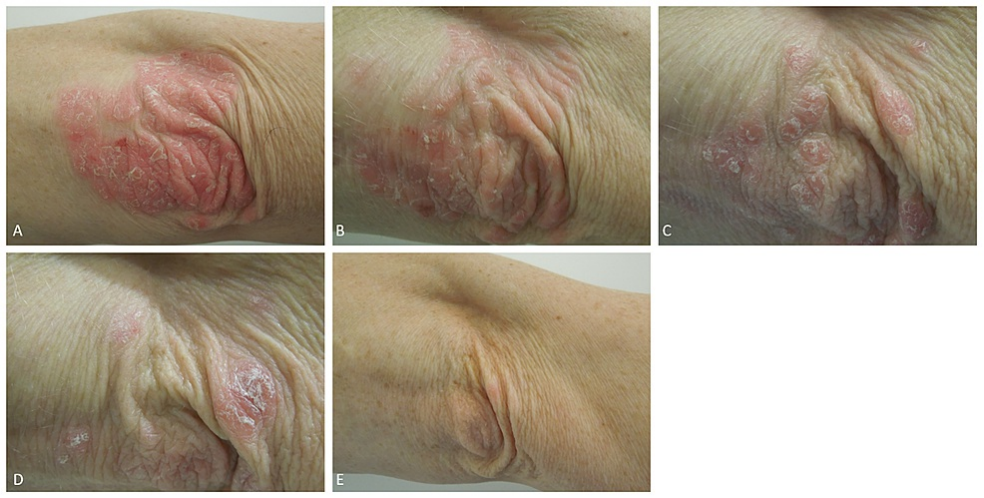
Progressive clinical improvement The images show lesion size before treatment (A), three weeks after (B), six weeks after (C), nine weeks after (D), and at the three-month follow-up (E)

Of note, 80% of all patients achieved a complete remission (PASI100) at the three-month follow-up. The remaining 20% reached at least PASI70. No adverse events were reported. In all cases, the lesion size decreased progressively (Figures 2, 3).

**Figure 4:**
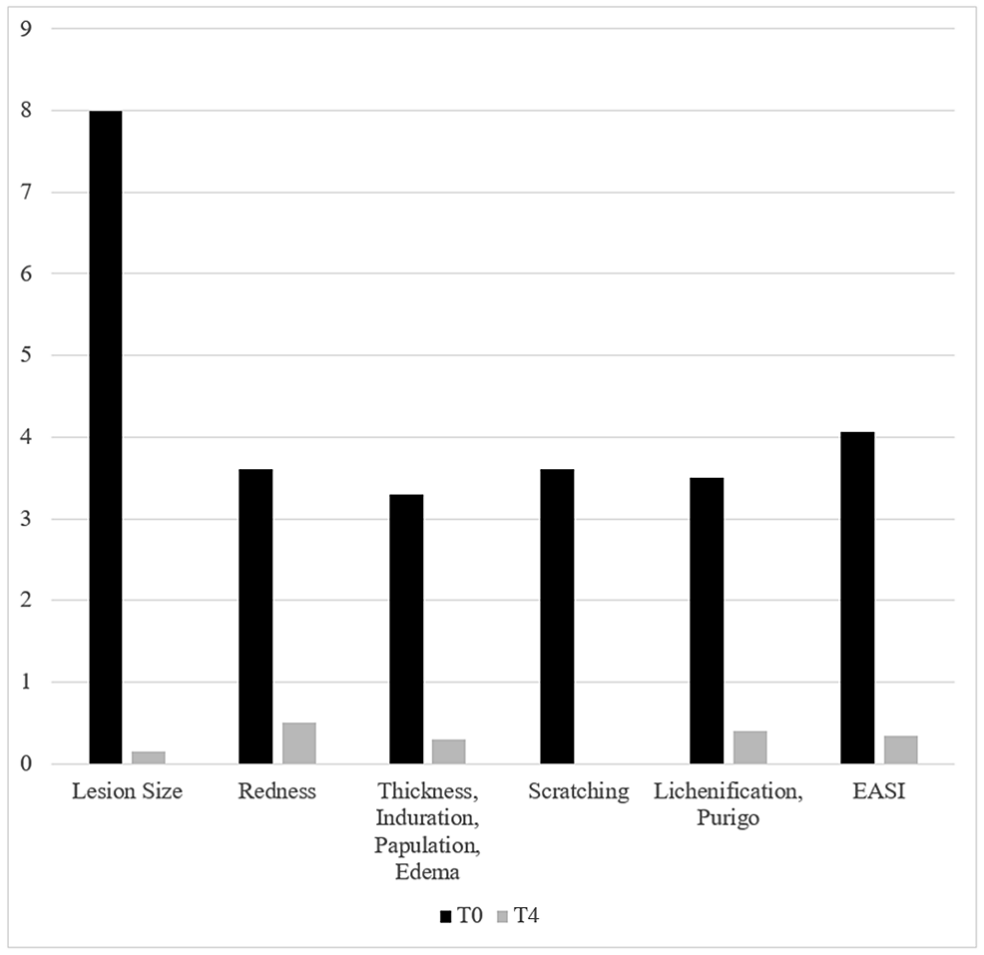
Signs of atopic dermatitis before treatment (T0) and improvement at the three-month follow-up (T4) EASI: Eczema Area and Severity Index

**Figure 5:**
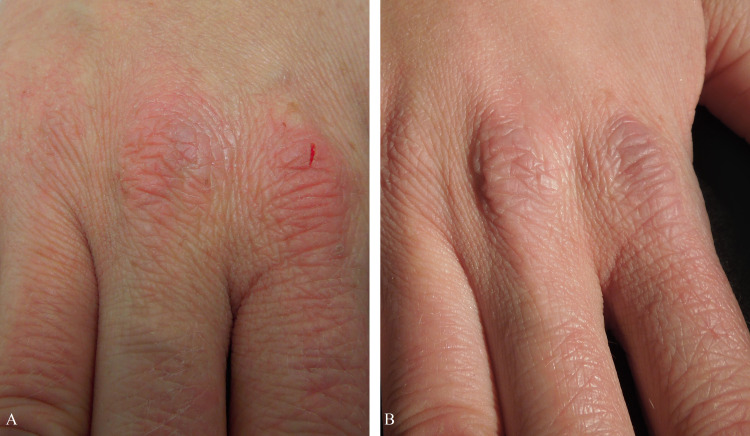
Reduction of signs of atopic dermatitis before (A) and after treatment (B)

A total of 10 patients with an average age of 22 years were treated for atopic dermatitis. Six of them were females and four were males. In six cases, efflorescences on patients’ arms (elbows/hands) were treated, and in four cases, patients’ legs were treated. The average lesion size decreased significantly from 8 cm² to 0.155 cm² (p<0.00001). Details such as clinical improvements and the significant reduction of the EASI (p<0.00001) are presented in Figures 4, 5.

**Figure 6:**
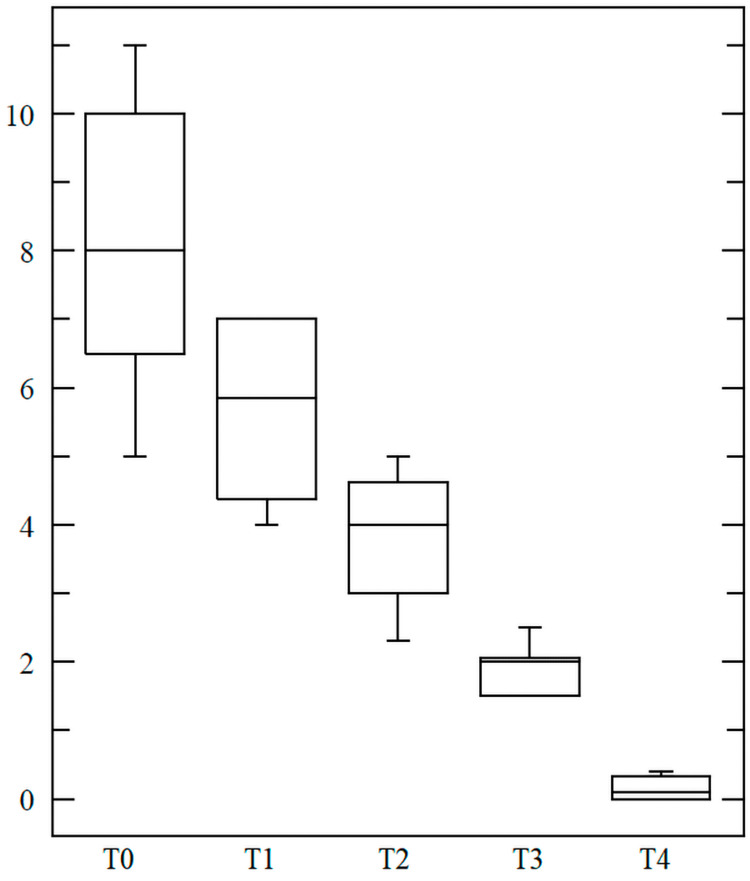
Progressive reduction in atopic dermatitis lesion size (cm²) The image shows lesion size before treatment (T0), three weeks after (T1), six weeks after (T2), nine weeks after (T3), and at the three-month follow-up (T4)

Significantly, 50% of all patients achieved a complete remission (EASI100) at the three-month follow-up. The other half reached at least EASI75. In all cases, the lesion size decreased progressively (Figure 6). No adverse events were reported.

## Discussion

For both psoriasis and atopic dermatitis, numerous new systematic treatment approaches have been established to reduce patients’ burden; however, there is still a need for limited courses where these treatment modalities are not useable. All systemic treatment options do have specific risks and benefits. Most compounds lead to suppression of the immune system; some suppress the hyperproliferation of keratinocytes [9]. Specifically, courses of limited but chronic plaque-type of psoriasis are difficult to treat. Topical therapies, such as vitamin D analogs and corticosteroids, represent a basic treatment modality for psoriasis [10]. In many cases, they are helpful but not powerful enough to obtain skin clearance [10].

Likewise, there are numerous treatment modalities to control atopic dermatitis. Clinical improvement and disease control with nonpharmacologic interventions such as emollient use, conventional topical therapies including corticosteroids and calcineurin inhibitors, and environmental and occupational modifications (when necessary) can be achieved for most patients [11]. If results are not satisfactory, additional phototherapy is recommended [12]. For those who do not respond adequately to the above-mentioned measures and in whom contact dermatitis has been considered, systemic immunomodulatory agents are indicated. Oral antihistamines and systemic antimicrobials can be beneficial under specific circumstances [12]. Monoclonal antibodies, which represent a modern treatment strategy, are not yet implemented in the current US guidelines though they are included in the recent European guidelines [13,14].

PRP is known to have anti-inflammatory and immunomodulatory effects [15-17]. The exact mechanism of action for the treatment of psoriasis and atopic dermatitis has not been reported so far. Chakravdhanula et al. have reported better outcomes in the PRP+MTX group vs. the MTX group alone. At week 16, all patients in their combinational therapy group achieved PASI50, 62.5% achieved PASI75, and 12.5% attained PASI90. None of the patients in the monotherapy group reached PASI50; however, they showed improvement in the range of 35-40% relative to baseline PASI [7]. Ghani et al. have reported improved biomarkers after the treatment of atopic eczema with PRP. Clinical examination revealed marked improvement and the patient reported marked control of itchiness and disappearance of rash [18]. Recently, Yosef et al. published encouraging results of their split-side study reporting a statistically significant reduction in the EASI score and Investigators' Global Assessment (IGA) score in the combined side [PRP plus narrowband-ultraviolet B (NB-UVB)] in comparison with the NB-UVB side [19]. Our results are encouraging too: 80% of all patients treated for psoriasis achieved complete remission (PASI100). The remaining 20% showed improvement in the range of 70-92% relative to baseline. Also, 50% of all patients treated for atopic dermatitis achieved EASI100. The other half showed improvement in the range of 80-94% relative to baseline. The diseases were controlled for at least 12 weeks. Age and gender had no impact on clinical results. All data were analyzed in an unblinded setting, and hence bias cannot be excluded. A larger sample size, longer follow-up, and a prospective study design including a control group would be desirable for future studies. Moreover, the optimal dosing and method of application should be investigated. The subdermal injection can be easily performed in localized small areas (e.g., the elbow); for larger areas, an application involving nappage technique with occlusion patches or liposomal cream might be a more practical approach.

## Conclusions

Our study revealed encouraging results concerning the treatment of psoriasis and atopic dermatitis with PRP. The autologous treatment was safe and effective in all patients, and no adverse events were reported. The lesion size, as well as PASI and EASI scores, significantly improved at the 12-week follow-up. More studies are required to validate these initial findings.

## References

[1] Chiesa Fuxench ZC, Block JK, Boguniewicz M (2019). Atopic Dermatitis in America Study: a cross-sectional study examining the prevalence and disease burden of atopic dermatitis in the US adult population. J Invest Dermatol.

[2] Takeshita J, Gelfand JM, Li P (2015). Psoriasis in the US Medicare population: prevalence, treatment, and factors associated with biologic use. J Invest Dermatol.

[3] World Health Organization. (‎2016 (2020). World Health Organization: Global Report on Psoriasis. http://apps.who.int/iris/handle/10665/204417/.

[4] Maisel-Campbell AL, Ismail A, Reynolds KA (2020). A systematic review of the safety and effectiveness of platelet-rich plasma (PRP) for skin aging. Arch Dermatol Res.

[5] Chen JX, Justicz N, Lee LN (2018). Platelet-rich plasma for the treatment of androgenic alopecia: a systematic review. Facial Plast Surg.

[6] Hu Z, Qu S, Zhang J (2019). Efficacy and safety of platelet-rich plasma for patients with diabetic ulcers: a systematic review and meta-analysis. Adv Wound Care (New Rochelle).

[7] Chakravdhanula U, Anbarasu K, Verma VK, Beevi SS (2016). Clinical efficacy of platelet rich plasma in combination with methotrexate in chronic plaque psoriatic patients. Dermatol Ther.

[8] Loibl M, Lang S, Brockhoff G (2016). The effect of leukocyte-reduced platelet-rich plasma on the proliferation of autologous adipose-tissue derived mesenchymal stem cells. Clin Hemorheol Microcirc.

[9] Menter A, Gelfand JM, Connor C (2020). Joint American Academy of Dermatology-National Psoriasis Foundation guidelines of care for the management of psoriasis with systemic nonbiologic therapies. J Am Acad Dermatol.

[10] Elmets CA, Korman NJ, Prater EF (2021). Joint AAD-NPF Guidelines of care for the management and treatment of psoriasis with topical therapy and alternative medicine modalities for psoriasis severity measures. J Am Acad Dermatol.

[11] Eichenfield LF, Tom WL, Berger TG (2014). Guidelines of care for the management of atopic dermatitis: section 2. Management and treatment of atopic dermatitis with topical therapies. J Am Acad Dermatol.

[12] Sidbury R, Davis DM, Cohen DE (2014). Guidelines of care for the management of atopic dermatitis: section 3. Management and treatment with phototherapy and systemic agents. J Am Acad Dermatol.

[13] Reynolds M, Gorelick J, Bruno M (2020). Atopic dermatitis: a review of current diagnostic criteria and a proposed update to management. J Drugs Dermatol.

[14] Wollenberg A, Barbarot S, Bieber T (2018). Consensus-based European guidelines for treatment of atopic eczema (atopic dermatitis) in adults and children: part II. J Eur Acad Dermatol Venereol.

[15] Zhang J, Middleton KK, Fu FH, Im HJ, Wang JH (2013). HGF mediates the anti-inflammatory effects of PRP on injured tendons. PLoS One.

[16] Bendinelli P, Matteucci E, Dogliotti G, Corsi MM, Banfi G, Maroni P, Desiderio MA (2010). Molecular basis of anti-inflammatory action of platelet-rich plasma on human chondrocytes: mechanisms of NF-κB inhibition via HGF. J Cell Physiol.

[17] Huber SC, De Lima Montalvão SA, Sachetto Z, De Paula EV, Annichino-Bizzacchi JM (2016). Autologous platelet rich plasma (PRP) provoke immunomodulatory response by an increase of regulatory T cells (CD4+ CD25+ FOXP3+) with a decrease of activated NK cells (CD16+ CD56+ CD69+) in a longitudinal study of patients with Behçet’s disease. Blood.

[18] Ghani R (2018). Platelet-rich plasma use in the treatment of eczema (atopic dermatitis): case report. Glob Sci J.

[19] Yosef A, Elkady N, Khattab F (2021). Possible clinical efficacy and tolerability of platelet-rich plasma with atopic dermatitis (Epub ahead of print). J Cosmet Dermatol.

